# HIV Testing for Children in Resource-Limited Settings: What Are We Waiting For?

**DOI:** 10.1371/journal.pmed.1000285

**Published:** 2010-07-20

**Authors:** Scott Kellerman, Shaffiq Essajee

**Affiliations:** 1 Center for Health Services, Management Sciences for Health, Arlington, Virginia, United States of America; 2 Clinton Health Access Initiative, Clinton Foundation, New York, New York, United States of America

## Abstract

Scott Kellerman and Shaffiq Essajee argue that the time has come to increase access to HIV testing for children, especially in sub-Saharan Africa.

**Summary Points**

- Expansion of prevention of mother-to-child transmission in resource-limited settings remains a challenge.
- In many countries, most HIV-exposed infants do not benefit from PMTCT programs, which results in a 30% or more transmission rate.
- Vertically infected infants not diagnosed in the context of PMTCT are rarely diagnosed until symptomatic with HIV, resulting in increased morbidity and mortality.
- Infant and pediatric testing programs are needed until PMTCT challenges are overcome or universal treatment of HIV-infected pregnant women becomes the norm.

## Introduction

**Figure 1:**
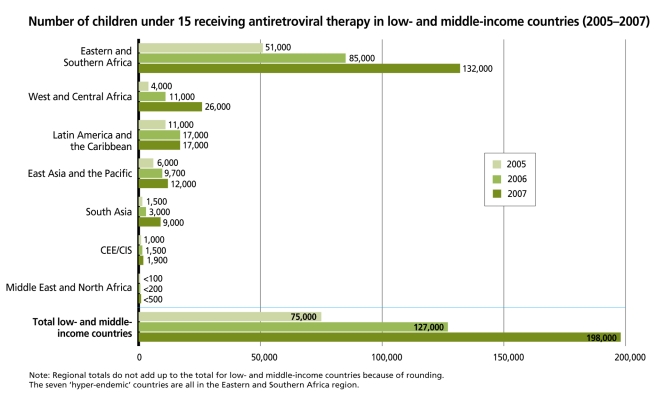
Number of children under 15 receiving antiretroviral therapy in low- and middle-income countries, 2005–2007. Source: UNICEF calculations based on data collected through the PMTCT and Paediatric HIV Care and Treatment Report Card process and reported in UNICEF Children and AIDS. 3^rd^ stocktaking report 2008, pp. 34–42 (http://data.unaids.org/pub/Report/2008/20081201_3rd_stocktaking_summary_en.pdf) [20]. Regions were recalculated according to UNICEF classification of regions.

In many African countries, HIV has reversed previously recorded declines in child mortality. Worldwide, children account for 18% of HIV-related deaths and 15% of HIV infections each year [1]–[3], an estimated 2.3 million children are infected, and 730,000 urgently need antiretroviral therapy (ART), which only about 275,000 currently receive. The mortality of untreated pediatric patients is very high in the first 2 years of life, and reaches 80% by age 5 [4]. While the number of children under age 15 in low- and middle-income countries receiving ART rose dramatically between 2005 and 2007 (Figure 1), it is nonetheless evident that those children currently on treatment still represent only a small proportion of those who need it. Coverage will need to be greatly expanded if the global community's goal of providing ART to 80% of children in need by 2010 is to be met [1].

As more low-cost fixed-dose combination antiretrovirals (ARVs) for children become available, the issue of access to medication is less of an impediment to treatment (Table 1). Why then are so few children in developing countries on ART? We propose that the primary reason is insufficient identification of infected children. There are many causes for this—including poor coverage of services for prevention of mother-to-child transmission (PMTCT), poor linkages to infant testing programs, provider uncertainty on how best to diagnose and treat infants, and insufficient numbers of pediatric HIV treatment sites—but the end result is that many infected children are either never identified or lost from the system before they can be enrolled into care. We believe it is essential for national HIV programs to recognize that HIV testing and counseling systems designed for adults do not meet the needs of children. The time has come to develop and implement specific strategies to increase opportunities for children to access HIV testing, especially in sub-Saharan Africa.

**Table 1:** Costs of Pediatric ARV for Resource-Limited Settings, 2009.

Pediatric Fixed Dose Combination	FDA Approved Date	WHO PQ Date	Cost per Year for a 10kg Child^a^	Per Pack Price^a^ (Pack Size)
D4T/3TC/NVP				
6/30/50	Aug 13 2007	Apr 23 2008	$60	$2.49 (60s)
12/60/100			$54	$4.54 (60s)
D4T/3TC				
6/30	Jun 19 2008	–	$48	$2.00 (60s)
12/60			$41	$3.42 (60s)
AZT/3TC/NVP	–	Oct 26 2009	$108	$4.50 (60s)
AZT/3TC	July 23 2009	May 25 2009	$80	$3.33 (60s)
ABC/3TC	Dec 19 2008	Oct 26 2009	$180	$7.50 (60s)

Based on ref. [21].

a Costs based on Clinton Foundation HIV/AIDS Initiative 2009 ceiling prices http://www.clintonfoundation.org/files/chaiarvpricelistaugust2009english.pdf.

As criteria for treatment initiation evolve and ART programs are scaled up in resource-limited settings, the need to expand HIV testing will become more urgent. Surveys in sub-Saharan Africa document 39% of adult men and women as having at some time been tested and received their results, up from 15% just 2 years before [3]. However, even when strong adult testing programs exist, access to pediatric testing remains low. The 2004 World Health Organization (WHO) HIV testing guidelines did not identify children as a specific target group for testing [5]. More recent WHO guidance on provider-initiated HIV testing provides direction on how to overcome barriers to testing children but offers little on how to operationalize pediatric testing [6].

Data from the South African CHER study highlight the survival benefit of early treatment for infants, showing an overall 75% decline in mortality in those infants who were started on ART immediately after diagnosis [7]. In response, the WHO has changed its treatment recommendations, calling for treatment of all infected infants under 12 months of age, irrespective of clinical stage [8]. This is a critical advance in treatment policy, which national AIDS control programs should adopt as soon as possible. But without better ways to identify infected infants, the policy alone will not change the treatment landscape in the short term. Although infant diagnosis is now available in many PMTCT programs, at current rates of PMTCT coverage, the majority of HIV-infected infants are born to mothers who were never tested and never received PMTCT prophylaxis. These infants are very unlikely to be identified and get on to treatment without targeted testing strategies. Scale up of testing programs for children will no doubt require investment in key areas such as training and support for providers, improvement of laboratory facilities and referral networks, and community mobilization, but such investments are necessary to reduce the substantial mortality of HIV in children.

Because of the marked survival advantage among those identified and treated in a timely manner, the US Centers for Disease Control and Prevention has recommended routine HIV testing for US adults during contact with medical facilities [9]. This represents a clear shift away from voluntary testing (which emphasizes personal choice) toward an emphasis on the public and individual health benefits of improved identification and control of HIV disease and prevention of HIV transmission. Of course, success in operationalizing these recommendations depends on a well-functioning health care system—which does not exist in many of the countries most affected by the AIDS epidemic. As such, these recommendations have not been widely implemented in the most affected parts of the world, where making a diagnosis is most critical, particularly in children.

## Building an Approach to Pediatric Testing

Current approaches to testing infants and children center on PMTCT programs. New approaches should build on the considerable success realized by PMTCT while its shortcomings are recognized. Routine testing of newborns may be an appropriate approach to identify infants missed by PMTCT programs, particularly in countries with high prevalence, while more targeted testing of infants and children at greater risk may be more cost effective for lower-prevalence countries. Regardless of the approach, there are significant challenges to testing children for HIV. In infants younger than 18 months, the persistence of maternal antibodies, the lack of appropriate laboratory facilities for PCR testing, the cost of assays, and the need to repeat PCRs in infants who are exposed to infected breast milk [10], make it difficult to implement infant diagnosis programs. WHO estimates that, in 2007, only 8% of infants known to be HIV-exposed were tested for HIV within the first 2 months of life [11]. Waiting for infants to develop symptoms or become old enough to test using standard rapid tests is not ideal but has become the norm in many places, resulting in children tested late in the course of their infection, when ART may be less effective.

Parental attitudes towards testing are important to ensure success, but anecdotal reports suggest that many parents are apprehensive about subjecting their children to HIV tests, especially when they are unsure of their own HIV status [12]. Equally important is the issue of what informed consent means for pediatric patients and their caregivers; the complexities of designing testing programs for children who neither seek out nor necessarily understand the consequences of a test; and the ethics of testing children who, if HIV positive, would indicate the mother's status as well. As pediatric testing programs are scaled up, it will be especially important to consider WHO guidelines which recommend that children be involved in the decision to be tested as much as possible, stressing that parental consent is always required and that the decision to test should be voluntary. Furthermore, in circumstances of extreme disadvantage, such as with orphans and vulnerable children, care must be taken to ensure that HIV testing does not cause harm because of the greater risk of discrimination and exploitation that these children face. This would require training and support for providers unfamiliar or uncomfortable with these situations.

Despite the inherent complexities, we believe that a focus on child testing apart from PMTCT is long overdue, beginning with national policies offering a multi-tiered approach to make pediatric HIV testing a routine element of care, and implementation support to make this a reality. We are not advocating universal screening of all newborns and infants, particularly in countries with lower prevalence, rather preferring more cost-effective, targeted approaches that consider higher diagnostic probabilities in different circumstances.

The following strategies, while not an exhaustive list, might result in improved access to testing. Given the very high mortality associated with HIV in children, even minimal attention to the development of a pediatric testing strategy might result in substantial decreases in morbidity and mortality.

## Entry Points for Testing

**Figure 2:**
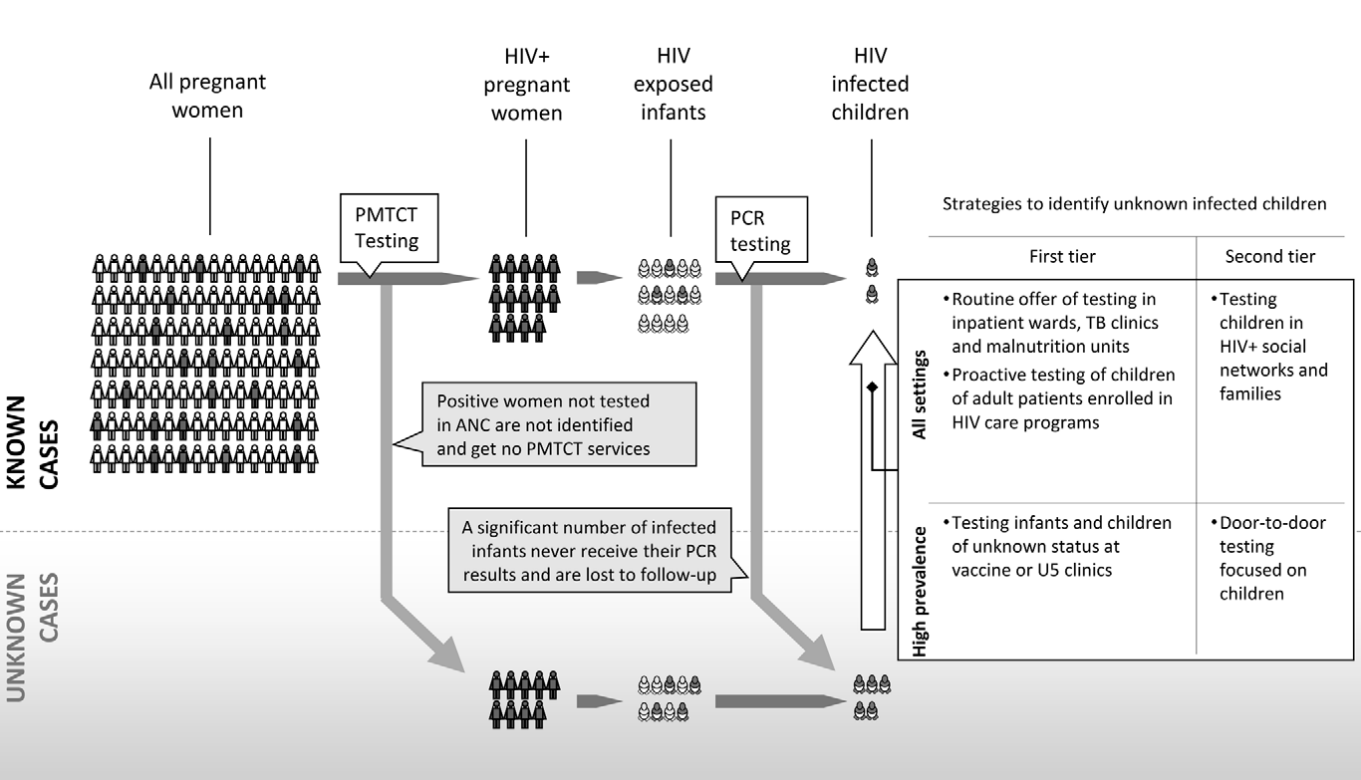
Despite improvements in PMTCT and infant testing coverage, many women and infants are not tested and are lost to follow up resulting in high infant and child mortality. Strategies to help increase the identification of infected children depend on the underlying seroprevalence and epidemiology.

Two groups of strategies that may be useful for case finding of children missed by PMTCT are presented here. First-tier strategies use existing systems to incorporate pediatric HIV testing into established entry points to care, whereas second-tier strategies require the development of new programs or systems to actively seek out and diagnose infected children and link them to care (Figure 2). First-tier approaches include variations of provider or program-initiated testing such as testing newborns when they present for immunizations—which may prove cost-effective in countries with high HIV prevalence. In such hyperendemic settings, an initial rapid test could be used as a screen to test mothers or their newborns, with a subsequent PCR for infants who test positive or whose mothers are positive. While such screening is potentially expensive, higher prevalence rates, and thus higher rates of diagnosis, may justify the increased costs. One study of routine testing in immunization clinics found that testing was well accepted and identified a large number of exposed children with an overall seropositivity rate of 10% [13]. In lower-prevalence settings, connecting the offer of testing to points of care where the concentration of infected children is likely to be higher such as pediatric inpatient wards, nutrition rehabilitation units, and tuberculosis clinics may be effective. In one recent report, 80% of parents accepted testing in pediatric inpatient wards, yielding a seroprevalence rate of 29% [14]. In Zambia, children admitted to the malnutrition ward were found to have high HIV prevalence rates (Marc Bulterys, personal communication). Medical settings, while an obvious point of contact, are not the only venues through which to reach affected children. Community organizations, especially those serving orphans or adults living with HIV, can also be important partners in expanding access to pediatric testing.

Second-tier approaches might include door-to-door or in-home testing, which may be especially useful for populations that are infrequent clinic attendees or simply lack access to care. Variations of social network testing, in which friends and acquaintances of HIV-infected persons or those at higher risk are targeted for testing, are promising strategies in US adults [15]. Such strategies could be adapted so that families affected by HIV are counseled to refer people within their families or networks, including children, for testing. Similarly, community-level interventions, such as contact tracing in which the entire family is offered testing if one family member tests positive, may prove valuable in developing countries. Data from Uganda found household-member and door-to-door testing strategies relatively effective and inexpensive as compared to stand-alone and hospital-based strategies [16]. To that end, a South African program, in which HIV-positive adults accessing ART clinics view a video in their local language encouraging them to have their children tested, resulted in increased uptake of pediatric testing in the region. Another approach, in which caregivers (e.g., grandmothers) collecting government checks are targeted with similar messages, has also shown promise [17],[18].

## Moving Forward

Many of the strategies proposed here have been tried and evaluated; however, implementing them in a coordinated fashion in resource-limited settings requires new investments. Provider-initiated testing in pediatric wards, routine testing of newborns and infants in immunization clinics, and door-to-door and family testing have all been attempted in sub-Saharan Africa. What is needed now is a more coordinated effort at the national level to ensure that infected children known to be exposed to HIV and those missed by PMTCT are identified and linked to care. Although challenging, especially when one weighs the parents' right to confidentiality against the child's right to care, a standardized approach to childhood testing is feasible. Indeed, in the US many states perform mandatory testing of newborns, allowing the clinician to offer postnatal ARV prophylaxis to the index case, comprehensive HIV care to the mother, and early treatment to the infected child, with resultant near-elimination of pediatric HIV mortality and mother-to-child transmission [19]. Finally, while the costs of establishing routine pediatric testing are not insignificant, they pale in comparison to the societal costs of delayed diagnosis and increased child mortality. Given the challenges of scaling up ART treatment services in resource-limited settings, we believe the targeted approaches described above may be a cost-effective, first strategy to decreasing the pediatric treatment gap in many countries and as with other prevention efforts, should be based on the local epidemiology of the epidemic. It is clear that new approaches and a coordinated response to testing children are necessary to close this gap. The global public health community should make this an urgent priority. Anything less is unacceptable.

## Abbreviations

ART: antiretroviral therapy
ARV: antiretroviral
PMTCT: prevention of mother-to-child transmission
WHO: World Health Organization
